# The Internet of Things in Health Care in Oxford: Protocol for Proof-of-Concept Projects

**DOI:** 10.2196/12077

**Published:** 2018-12-04

**Authors:** Edward Meinert, Michelle Van Velthoven, David Brindley, Abrar Alturkistani, Kimberley Foley, Sian Rees, Glenn Wells, Nick de Pennington

**Affiliations:** 1 Healthcare Translation Research Group Department of Paediatrics University of Oxford Oxford United Kingdom; 2 Global Digital Health Unit Department of Primary Care and Public Health Imperial College London London United Kingdom; 3 Oxford Academic Health Sciences Network Oxford United Kingdom; 4 Oxford Academic Health Sciences Centre Oxford United Kingdom; 5 Oxford University Hospitals NHS Foundation Trust Oxford United Kingdom

**Keywords:** Internet, computer systems, computing methodologies, information systems, information storage and retrieval, dataset, patient care, health services, Internet of Things, Internet of Medical Things

## Abstract

**Background:**

Demands on health services across are increasing because of the combined challenges of an expanding and aging population, alongside complex comorbidities that transcend the classical boundaries of modern health care. Continuing to provide and coordinate care in the current manner is not a viable route to sustain the improvements in health outcomes observed in recent history. To ensure that there continues to be improvement in patient care, prevention of disease, and reduced burden on health systems, it is essential that we adapt our models of delivery. Providers of health and social care are evolving to face these pressures by changing the way they think about the care system and, importantly, how to involve patients in the planning and delivery of services.

**Objective:**

The objective of this paper is to provide (1) an overview of the current state of Internet of Things (IoT) and key implementation considerations, (2) key use cases demonstrating technology capabilities, (3) an overview of the landscape for health care IoT use in Oxford, and (4) recommendations for promoting the IoT via collaborations between higher education institutions and industry proof-of-concept (PoC) projects.

**Methods:**

This study describes the PoC projects that will be created to explore cost-effectiveness, clinical efficacy, and user adoption of Internet of Medical Things systems. The projects will focus on 3 areas: (1) bring your own device integration, (2) chronic disease management, and (3) personal health records.

**Results:**

This study is funded by Research England’s Connecting Capability Fund. The study started in March 2018, and results are expected by the end of 2019.

**Conclusions:**

Embracing digital solutions to support the evolution and transformation of health services is essential. Importantly, this should not simply be undertaken by providers in isolation. It must embrace and exploit the advances being seen in the consumer devices, national rollout of high-speed broadband services, and the rapidly expanding medical device industry centered on mobile and wearable technologies. Oxford University Hospitals and its partner providers, patients, and stakeholders are building on their leading position as an exemplar site for digital maturity in the National Health Service to implement and evaluate technologies and solutions that will capitalize on the IoT. Although early in the application to health, the IoT and the potential it provides to make the patient a partner at the center of decisions about care represent an exciting opportunity. If achieved, a fully connected and interoperable health care environment will enable continuous acquisition and real-time analysis of patient data, offering unprecedented ability to monitor patients, manage disease, and potentially deliver early diagnosis. The clinical benefit of this is clear, but additional patient benefit and value will be gained from being able to provide expert care at home or close to home.

**International Registered Report Identifier (IRRID):**

DERR1-10.2196/12077

## Introduction

### The Use of Internet Devices to Capture Health Data

The popularity of smart watches, wearable tech, and mobile phone technology have ushered an era of internet-enabled digitally connected devices. Daily, one can observe wide use of mobile phones and relatively low-cost devices and associated internet connectivity. Mobile phone user numbers are increasing worldwide; 95% of individuals in the United States and 94% of individuals in the United Kingdom own a mobile phone, with the majority of them having access to mobile phones [1,2]. Global spending on these devices is projected to be US $410 billion in 2022 [3]. In 2017, there were more connected devices (estimated 8.4 billion) than people (around 6 billion) in the world [4]. The number of devices is estimated to grow to 20.4 billion by 2020 [4]. These devices are used to track steps, heart rate, and various forms of activity and health data, demonstrating that consumers are becoming increasingly interested in their wellness and health [5] (also known as the *quantified self*-movement [6]).

### Defining the Internet of Things

The Internet of Things (IoT) or Internet of Medical Things (IoMT) extends the Web through the deployment of ubiquitous devices with capabilities for embedded identification, sensing, and data exchange features [7]. These smart objects form the foundation of cyber-physical networks, which establish an extensible foundation for a networked data exchange [8]. IoT enables the capability to deploy, manage, and analyze interconnected devices, constructing a real-time extension to provide broad sensory analytic hardware and software for our physical world.

### The Possibility of Internet of Things to Address Health Care Challenges

IoT has potential to collect and integrate possibly more precise, relevant, and high-quality data in real time to monitor processes and outcomes [9]. IoT can contribute to connecting patients, medical staff, wearables, information technology systems, and medical equipment and integrating them using on-demand internet connectivity [10]. The most promising benefits of IoT for health care might be in increasing workforce productivity, cost savings [11], operational efficiencies [12], improved patient experience and care, and human error reduction [13,14]. These benefits can be applied to address health care challenges and facilitate behavior change and promote well-being of patients by giving them ownership of their health (Textbox 1; the textbox is adapted from Table number 1 in Meinert et al’s analysis [15]). Such ownership could enable proactive behavior changes, preventing onset of health issues, and better management of existing conditions.

IoT offers opportunities to take the model of health care from encounter-based care through connected continuous care. The rapid growth of consumer-centered health-orientated apps and wearables has created an exponential expansion of device data collection. This growth has encouraged the development of patient-centered focus in IoT. These widely used connected devices could enable improvements in patients’ safety by integrating different sources of data.

**Key health care Internet of Things (IoT) use cases.**

Improving patient safety and outcomes by

- Using insights from the integration of different types of data (eg, health and environment) collected by different sources (eg, mobile devices and electronic health record)
- Using data for improved prediction (eg, artificial intelligence)

Facilitate people’s lives and reduce costs by

- Providing tools such as voice-assisted note taking and reminders
- Automating processes that do not need humans

Improving health care delivery by

- More actively involving people in their health care decisions
- Providing the right information at the right time
- Giving access to health care like other industries such as banking and retail industry do (eg, Amazon) by providing teleconsultation, ePrescribing, and delivery of medication

This can then be used for proactive identification of needs to enhance delivery by providers and optimizing value for payers by identifying gaps that can be closed. Moreover, patients could be more effectively engaged in taking care of their own health, which could facilitate prevention and monitoring of illnesses.

### Current Trends in Industry and Research

To inform perspectives on the IoT, we have drafted a systematic review protocol to analyze the state of the literature of the technology and its applied use in health. This protocol and the review will be submitted for peer review in a medical journal. This section summarizes initial findings on current trends in the industry and research.

### Internet of Things Properties

Having defined IoT as devices that create a framework for data exchange and sensory analysis of the physical world, we further refine their implementation characteristics of their use. IoT devices follow 7 physical properties in their design and implementation [16]:

1. Tracking: The capability to capture geophysical and dimensional properties in movement. This tracking enables awareness of location, movement, and trends and provides proximity to activity [16].
2. Identification and authentication: Correlation and authentication of devices to individuals is vital to ensure linkage of data to a specific individual. Such identification and authentication enable integrity and verification of source data [16].
3. Data collection (monitoring): The capability to store, retrieve, and send data for analysis [16].
4. Sensing: The capability to allow for awareness of physical presence [16].
5. Control: Ensure that the behavior of a person or machine is desired and allows to take corrective action [17,18].
6. Optimization: Using rules to enable complex decision making by the system or sensors to improve performance [17,18].
7. Automation: Combining control and optimization of data and control from IoT and other sources to allow making independent decisions [17,19].

The IoT provides broad app scenarios based on these core features as they create the ability for data collection, which can be structured via capabilities enabled in software design, using tracking and sensing to create proximity and awareness data. These IoT features can vary in their complexity from rudimentary and complicated to highly complex [17].

### Internet of Things in Health and Care

The rapid growth of consumer-centered health-orientated apps and wearables has created a significant expansion in device data collection. This growth has encouraged the development of patient-centered focus in IoT. The equal challenge and opportunity in this application of IoT in health is to create a structured framework for both use and data transfer to allow data to be usable across social, clinical, and wider health care contexts to extend the reach of primary, secondary, and tertiary care.

### Health Internet of Things Classification and Use Cases

Health IoT can be beneficial from the perspective of the patient because these devices create a means of directly connecting health services with the patient. Health IoT use cases can be broadly categorized in the following patient-centered contexts [7,9,14,20,21]:

1. Safety: real-time health data management enables health systems to enable individual and population markers to enable patient safety. Specific cases include:
  - Vital signs and patient monitoring,
  - Real-time health status and predictive information,
  - Public health policy and regulation decisions in pandemic scenarios,
  - Pharmacoepidemiology, and
  - Broad population health management.
2. Satisfaction: traditionally, interactions with health systems require physical visits to primary or secondary care for capture of health-related data. The use of IoT enables an extension of data and the ability to provide health system interaction outside of formal health care settings. The convenience of providing this type of care may influence patient satisfaction. Specific cases include:
  - Responsive feedback and
  - Telemedicine.
3. Engagement: building on a health record, the ability to coordinate decision making, create patient-specific education, and manage prescriptions, all enable an interactive virtual basis for information exchange between providers and patients. Specific cases include:
  - Care coordination,
  - Medicine adherence, and
  - Personalized health care and well-being planning.

Although it might be possible to examine health IoT from a classification context removed from the patient, a patient-centered view is essential in consideration of the way health IoT can enable an expansion of health systems beyond the physical parameters held in traditional implementation of care. By using such a view to place health IoT use cases, we can begin to envisage both the opportunities and the challenges in the deployment.

### Key Implementation Challenges

Although the implementation of IoT devices has broad reach and capability, their use has identified key issues in their deployment [5]:

1. Computational limitations: Devices, due to a limited form factor are constrained in their on-board computational power.
2. Energy limitations: Another limitation of form factor is the amount of battery storage that can be stored to power devices. Limitations in battery storage means devices have to be recharged for ongoing data transmission, which can limit the utility of devices, especially in scenarios of intense data capture.
3. Scalability: Systems storing data must be able to increase capacity; as the number of connected devices increases, this places increased demands on network infrastructure and computational infrastructure to process and manage inbound data.
4. Multiplicity of devices: With open frameworks, there creates a possibility to design an infinite number of devices, but the challenge with these devices is the need to validate the data they are providing and support their use. Device manufactures may also ignore standards and develop proprietary data structures inhibiting interpretation by third parties.
5. Dynamic network topology: Although the ability to create a dynamic network structure is an advantage of IoT systems, the permutations of the network design create a need to ensure there is ability to analyze each data point, which may be difficult.
6. Tamper-resistant packages: Because devices are implemented broadly and not within closed networks, the potential ease of access and ability to compromise these devices is a key security concern. To mitigate the risk of tamper, it is necessary to develop tamper-resistant form factors that cause compromise in system functionality and capability to achieve integrity of devices. In the event devices are compromised, the ability to detect this to report potentially compromised data is vital.
7. Dynamic security updates: Another key consideration of security is the need to ensure integrity of embedded systems; the expansive proliferation of IoT devices creates a possibility of exploiting systems for security penetration, and IoT can enable dynamic updates of devices.

### Case Studies

In this section, we explore current implementation use cases of IoT technologies. These technologies were selected following review of technologies presented at Health Care Information and Management Systems Society (HIMSS) 2018 and, additionally, identified following review of exemplar technologies from initial data analysis in a forthcoming systematic review on IoT in medicine. Although this list is not comprehensive, it provides examples of implementation use cases impacting broad elements of care. As part of this study, further review of available technologies matched to areas of user need shall be identified. Key considerations within these technologies include technical aspects of solution implementation and their benefits from a patient perspective.

### Medically Prescribed Internet of Medical Things Devices: Livongo

#### Challenge

Managing chronic conditions is challenging for patients because they require medication adherence and lifestyle modifications [22,23]. Increasingly, patients are also managing multiple chronic conditions, with about two-thirds of adults living with 2 or more chronic conditions and the prevalence increasing up to 70% among the adult population aged over 70 years [24].

#### Opportunity

Livongo provides a platform for management of chronic conditions, through use of a digitally connected software ecosystem for device interactivity and real-time care support through consumer health technology. By using artificial intelligence (AI) to learn patterns and create actionable insights, Livongo is able to store real-world data on patient history for analysis and action [22].

#### Use Case or Patient and User Perspective

Livongo's diabetes program enables an IoT glucose monitor to send data directly to its cloud infrastructure, allowing for real-time data analysis and support. This enables the capability for real-time patient feedback and support throughout the day. Simplifying means of data collection into accessible devices and tracking status via interactive dashboards enable better monitoring and feedback for patients and provide capabilities for better remote clinical feedback. The device is *prescribed* to appropriate patients by their health provider and feeds data back to the provider’s electronic record system. This system has been selected as a preferred supplier for the Cerner Millennium (Kansas City, USA) electronic record system that is in use at Oxford University Hospitals (OUH) National Health Service (NHS) Foundation Trust [22].

#### Evidence and Outcomes

Livongo is collaborating with the company Lilly to identify trends in the impact of remote diabetes self-management education, detailing the drivers of healthy behaviors and helping our understanding of how people living with diabetes can stay more engaged in their health [22]. Analysis of real-world behavior studies will provide evidence on what impact use of this system has on disease management. In addition, a study found that individuals with diabetes had lower likelihood of having hypoglycemia and hyperglycemia when using Livongo with a *diabetes coaching team* [23].

### Environmental Monitoring: CleanSpace

#### Challenge

Evidence suggests that air pollutants may cause serious health effects and specific population groups are especially sensitive to the impact of negative air quality [25,26]. Although tracking of air quality is done at a macro level, detailed hyper-local data are difficult to capture because of a lack of sensors collecting this information [27].

#### Opportunity

CleanSpace is an IoT sensor used to monitor air pollution. It uses machine learning to create hyper-local population data to enable users to understand in real-time quality of air. The sensor harvests radio-frequency energy from its environment to recharge its battery. At present, it only measures carbon monoxide (CO) but is claimed as a generic marker of other pollutants (particularly from traffic) [28]. CleanSpace is a product of Drayson Technologies who already have an academic and commercial relationship with the University of Oxford and OUH.

#### Use Case or Patient and User Perspective

The small form factor of CleanSpace enables them to be located in buildings or on mobile physical objects (eg, bicycles) to generate low-level data on air pollution. These data can be used by researchers, health care providers, and public health services to analyze trends on possible correlation of air pollution and health conditions. Awareness of patient-level pollution can lead to individual action in seeking activities or events where there is better air quality, encouraging actions to promote better air quality within the individual’s environment, and potential to instigate preventative treatment of respiratory disease.

#### Evidence and Outcomes

The Environment Research Group (King's College London) and National Physical Laboratory completed product performance validation measuring ambient concentrations of CO, comparing CO with other major urban pollutants.

### Bring Your Own Device Integration: Validic

#### Challenge

There are huge challenges for access, integration, standardization, storage, and use of IoT device data. As increased number of devices are brought to market, a need to leverage these products within a common software framework will be necessary to leverage the opportunity to have continuous data linkage among devices. This is particularly true with the proliferation of personal health monitoring devices. These devices have the potential to contribute to a *bring your own device* (BYOD) IoMT infrastructure, but only if the data are appropriately curated.

#### Opportunity

When device connectivity and data access can be simplified, this can enable deriving and analyzing meaningful insights. Interactions between health care providers and their patients can be facilitated, which can improve patient adherence and engagement and can be used to more effectively manage multiple conditions and programs of care.

#### Use Case or Patient and User Perspective

Validic Mobile [29] is a set of libraries that enables health care organizations to easily integrate Bluetooth smart in-home health devices (eg, blood pressure monitors, thermometers, and heart rate monitors), Apple Healthkit [30], and VitalSnap technologies [31] into their existing iOS and Android mobile apps. The VitalSnap technology allows access to data from nonconnected devices using the camera on the mobile phone to capture and record data from the device’s display screen, which is validated by the device user.

Validic Impact is a remote monitoring device-agnostic platform that operates between devices and software on mobile devices, laptops, and personal computers. Validic Impact involves device connectivity and data delivery, customizable rules engine and alerts, patient enrollment flows, patient onboarding and support tools, clinician ordering or monitoring dashboard, integration into the clinical record, remote care admin dashboard, and device management tools.

Validic has been selected as the BYOD partner of choice for the Cerner electronic health record (EHR) system that OUH uses.

#### Evidence and Outcomes

Validic enables real-world data, which can be used to validate app claims by creating a single-point connection to perform analytics on individual- and population-level use.

### Voice-Enabled Devices: Orbita

#### Challenge

Amazon Alexa, Google Assistant, and multiple other chat bots provide capabilities to intuitively communicate with users via voice. Although they provide the capability for active user engagement, their base functionality requires regulation and customization to have functional use in health contexts.

#### Opportunity

Using application programming interfaces (APIs), voice-enabled systems can integrate with EHRs and combine data from connected devices. This data ecosystem can create surveys, capture results, manage common requests and questions, and use AI to provide automation of these interactions.

#### Use Case or Patient and User Perspective

Orbita voice [32] is working with care homes to provide the ability to give families the ability to interact with each other via voice, give clients the ability to be able to ask questions about medications, clarify postsurgery instructions, and develop other bespoke scenarios that mirror existing face-to-face interactions. Having the capability to increase communication and engagement on treatment and better data collection on issues impacting care delivery could lead to stronger patient engagement and treatment adherence through continuous communication channels.

#### Evidence and Outcomes

There is increasing data to indicate the rise in popularity of using voice-enabled response; however, the development of these technologies and their implementation use cases are nascent and require further examination.

### Personal Health Records: Apple Health Records

#### Challenge

The vendor-specific nature of medical data capture and concerns for privacy and security make access to personal health data stored by providers challenging for patients [33]. Viewing this information is often complicated (requiring multiple log-ins or storage credentials). Recently *patient portals* have been developed, but these typically only provide Web-based access to individual provider systems [34].

#### Opportunity

Mobile devices have mass consumer appeal, the ability to create a user-friendly, secure, and integrated means to capture and integrate health data with a platform, which can be shared with clinical teams. As patients move from health systems to using consumer devices that record medical-related information, the ability to integrate these data in one place creates a scalable and transportable mechanism for storage of health data.

#### Use Case or Patient and User Perspective

Using the Fast Health Care Interoperability Resources (FHIR), standard for data sharing of electronic medical records [35], plus the Substitutable Medical Apps, Reusable Technology (SMART) infrastructure for secure access [36], a pilot group of medical centers in the United States is enabling patients to access their medical data via the Apple Health Records feature available in Apple iOS version 11 [37]. APIs to enable SMART on FHIR app use are being deployed at OUH in the third calendar quarter of 2018—the first UK site to do so.

#### Evidence and Outcomes

There is a growing body of studies examining the portability and use of Apple Healthkit for patient uptake and adoption [38]. However, these systems require Apple hardware accessibility, and this increases the challenge of vendor lock-in. Although the utility of a general health data management system independent from hospital EHRs is intuitive, its application for adoption requires further study.

### Wayfinding: Kaleida Health

#### Challenge

Kaleida Health [39] serves 8 counties in New York with an average of 17,000 visitors per day and more than 1 million patients annually. Visitors are likely to ask an employee for directions if are not given direction every 30 feet while traveling through a building. This can increase anxiety of visitors, interrupt staff, and decrease productivity [40].

#### Opportunity

It is estimated that by 2019, 25% of health care organizations will use experimental wayfinding [41]. The goal of this study was to implement a wayfinding system that helps daily visitors confidently navigate Kaleida’s campuses. The purpose of the wayfinding tools was to provide a convenient end-to-end experience. The potential benefits of implementing a wayfinding system include understanding where users are going, cutting wait times and improving on-time appointments, reducing staff interruptions, balancing demand and capacity, improving customer satisfaction and the patient experience, lowering stress, and differentiating from competitors.

#### Use Case or Patient and User Perspective

Kaleida implemented a number of wayfinding tools, including indoor Global Positioning System and mapping, Bluetooth Low Energy beacon technology, mapping apps (eg, Google Maps), location- and condition-sensing technologies and platforms, IoT aggregation platforms, contextual messaging, parking management systems, digital signage, and self-service kiosks. Training was provided to make visitors and staff aware of the wayfinding tools. The experience for visitors starts at home with appointment notifications on their mobile phone, driving directions to the parking lot closest to the location of their appointment, suggestions on when to leave home, and integration with the city’s parking system and other parking garages. Inside the hospital, an internal geofence shows a 3D map of the destination to the visitor with a blue dot moving along the route to indicate the visitor’s current location. Secure connections are provided that link back to the patient’s electronic medical records.

#### Evidence and Outcomes

Key benefits of the wayfinding solution are improved efficiency, patient satisfaction, and engagement. The solution provided an integrated experience to visitors.

### Patient and Public Involvement

As demonstrated by the case studies, IoT technologies herald promise for stronger data integration, means to encourage patient proactive health monitoring, data integration, and other benefits. To better understand these considerations, factors influencing user perspectives on the impact of these technologies, and needs associated with distributed device accessibility, we propose to hold focus groups with patients to further explore the impact these technologies could have on their experience of care.

An application to hold these focus groups has been submitted to the University of Oxford’s Central University Research Ethics Committee. This study will be conducted with support from the Patient and Public Involvement, Engagement and Experience team at the Oxford Academic Health Science Network.

If the projects proposed earlier are chosen, it will also be possible to conduct a service evaluation of technology use at OUH, with the patients and professionals involved.

### Implementing Internet of Things at Oxford

#### Background of Oxford’s Digital Health Infrastructure

Oxford’s health care is delivered by 3 core providers:

1. OUH, an approximately 1200-bed acute hospital over 4 sites in Oxford (John Radcliffe Hospital, Churchill Hospital, and Nuffield Orthopaedic Hospital) and Banbury (Horton General Hospital), which provides a full spectrum of emergency and elective physical health services for the city, a regional population of up to 3.5 million, and some national services (eg, Craniofacial surgery).
2. Oxford Health (OH) NHS Foundation Trust, which provides in- and out-patient mental health service across Oxfordshire and community health services across a wide geographical footprint, including the neighboring counties of Berkshire, Buckinghamshire, and Wiltshire.
3. Primary care services are delivered to the 740,000 patients of Oxfordshire by 70 general practices (GP).

The majority of primary and secondary care services are commissioned by the Oxfordshire Clinical Commissioning Group (CCG). In addition, the South Central Ambulance Service (SCAS) provides emergency response and transportation services and Oxford County Council (OCC) provides social care.

Oxfordshire has a relatively mature digital health care ecosystem. This has recently been acknowledged by the award of Global Digital Exemplar (GDE) status to OUH, OH, and SCAS. Collectively, this has injected £33.4 million of funding (including match) into developing services to reach a standard that is comparable with the best international health care institutions. At present, each of the provider institutions has a different EHR provider: Cerner at OUH, Adastra and CareNotes at OH, Egton Medical Information Systems (EMIS) Health at all bar 2 GP practices, Liquid Logic at OCC, and 3 separate systems at SCAS covering out-of-hours, and 111 and 999 services.

### Innovation

OUH and the Oxford Academic Health Science Network have received funding from the European Union’s European Regional Development Fund to support the delivery of a digital health innovation program called TheHill. This funding is being matched by OUH and runs from April 2018 for 2 years. It will enable the employment of staff plus events and seed funding to support the development of needs, ideas, and businesses who are working on innovative digital health solutions. In addition, an innovation hub is being built on the John Radcliffe Hospital campus in partnership with the School of Architecture at Oxford Brookes University. Although the activities are funded and sited on the OUH site, TheHill will support ideas from across the Oxfordshire health ecosystem. It is intended that research, development, and evaluation activities will take place in partnership with all members of the Oxford Academic Health Science Centre (AHSC), including Oxford University.

### Challenges

Local challenges in the implementation of new digital systems exist but are surmountable and outweighed by the opportunities that exist in the Oxford ecosystem:

- There is heterogeneity of clinical systems reflecting a current lack of digital connectivity among the providers, with regard to the electronic medical records that are used by frontline clinicians to treat patients. Some integration exists through the Oxfordshire Care Summary—a core subset of clinical information that is available to members of OUH, OH, GPs, and SCAS.
- The *patient-facing or self-management* work-stream of the OH GDE Strategy mentions the use of consumer wearable sensors. The OUH and SCAS GDE roadmaps do not explicitly refer to the use or integration of IoT data.
- At present, there is no clear guidance for health care workers on how to engage with IoT devices (eg, which ones to recommend to patients and how to use them).
- There are general concerns from health care staff about data security and privacy when using or accessing patient data. The European Union General Data Protection Regulation and UK-specific Caldicott guardian infrastructure provide frameworks but require experience and expertise to apply to new technologies and systems.
- Resources (staff, support, and capital expenditure) for the implementation of new digital systems in the NHS are limited, although the GDE programme provides a window of opportunity.

### Population Health

Digital population health involves the integration of data across a patient’s interactions with health and care services to create a longitudinal care record. This can enable better coordinated, more effective, and efficient care for both the individual and the population they are part of. It is often extended to include any aspect of the health record that extends engagement with patients, for example, through patient portals and personal health records.

The OUH GDE programme is funding the procurement of a set of digital population health solutions. The implementation of these solutions is being supported by the CCG through their technical support team at the South Central West Commissioning Support Unit. These products are the key conduits to enable IoT integration, and therefore, we will focus on how this might be achieved.

The products that Oxford is implementing are provided by Cerner Inc (Kansas City, USA). This means that integration to OUH’s EHR is embedded but the solutions are vendor agnostic, so integration to the other providers in the network will be possible without the need for them to change their EHR systems.

Three technical solutions will be implemented:

1. Health information exchange: This is the ability to view an aggregated summary of the data held in the EHR of all connected providers for an individual patient. It is planned to go live in October 2018. The information shown includes vital signs, and so, this will be a potential mechanism to surface personal connected device data to all health care providers regardless of the provider EHR through which it is ingested.
2. HealtheIntent: This is cloud-based, programmable data platform that receives and normalizes patient-level data from multiple health sources, such as an EHR, but can also include complementary data reflecting the social determinants of health (eg, environmental monitoring and economic factors). This will go live in February 2019. Once the data exist in the system, a variety of off-the-shelf (HealtheRegistries) and bespoke (HealtheAnylytics, HealtheDataLab, and HealtheCare) tools can be used to interrogate, analyze, and draw actionable insights from the data.
3. HealtheLife: This is the patient portal for OUH. It will go live in December 2018. It provides the functionality for patients to view aspects of their health record and is the potential mechanism through which consumer IoT data could be connected or prescribed IoT device data presented to patients. It only interacts with the OUH EHR (Cerner Millennium) and so does not show or interact with data held in HealtheIntent. Separate portals exist into each EMIS instance for users to see their primary care records and OH plans to open its own portal toward the end of 2019, although currently the functionality has not been confirmed.

Importantly, Oxford will be the only second UK site to go live with all these solutions.

A regional consortium will be applying for funding from NHS England’s health data interoperability project, the Local Health and Care Records Exemplar. This offers additional opportunities for proof-of-concept (PoC) studies to be undertaken. The consortium may feature industry partners offering cloud-hosted solutions that integrate tools to apply machine learning to the population health data that are collated.

**Figure 1:**
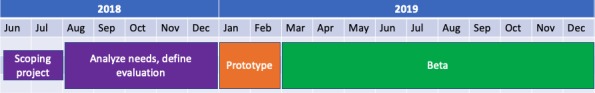
High-level project Gantt chart.

### Additional Technical Platforms

#### Substitutable Medical Apps, Reusable Technology on FHIR: Fast Health care Interoperability Resources

SMART is an open, standards-based technology platform that enables innovators to create apps that seamlessly and securely run across the health care system. It uses the FHIR. It is rapidly becoming accepted across the digital health community as the default standard for developing apps that can run across different vendor platforms, without requiring the creation of point-to-point data exchange solutions for every implementation.

OUH will be the first UK site to implement Cerner’s Ignite APIs that enable SMART apps to read and write data to the EHR. Once HealtheIntent is implemented, the Ignite APIs will provide similar access to this resource. These APIs will be live in Oxford from Q3 2018. SMART apps can be developed internally or procured from an increasing number of small and medium size enterprise vendors (mostly based in the United States) who are publishing them to an *app store*. New apps will require a combination of validation from Cerner plus at a local level. The processes to support this are evolving.

#### CareAware

CareAware is Cerner’s platform for integration of IoMT data. It is cloud hosted, EHR agnostic, and vendor neutral. It is currently deployed in Oxford. It is enabled by a library of over 1000 of the most common medical devices. A validation program exists to onboard additional IoMT devices. However, 2 different routes exist to feed data into the system depending on whether they are prescribed to consumer, also known as BYOD; this is shown in Figure 1:

1. Prescribed devices are typically linked through a full service from the vendors. Two key partners have established relationships with Cerner to do this: IdealLife and Livongo. Within the US market, both can be drop shipped via an order placed in the Cerner Millennium EHR. Depending on the service contract, the provider will ship their devices, onboard users through proactive calls, and provide full ongoing technical service.
2. Cerner has partnered with Validic to ingest and normalize data from BYOD devices. This is initiated by patients through the HealtheLife portal, where the device can be selected and permissions granted to access the personal data stored by the device vendor.

### New Device Research

Cerner’s *Project Yukon* is an experimental research project aiming to create a *connected* medical examination room. This will feature voice, video, and movement trackers; integrated examination devices (such as stethoscopes); and point-care-testing (eg, blood and urine analysis). It has some similarities to the consumer shopping experience that Amazon has recently demonstrated.

The potential to on-board data from more advanced diagnostic devices, such as the Firefly otoscope (used to examine the ear canal), provides opportunities to use IoMT to perform more accurate remote diagnosis and management. This could complement existing voice and video telehealth systems to deliver more responsive care [42]. Needs exist where patients and professionals are geographically remote from each other. This may support care where patients are mobile or time constrained or where specialist expertise only exists in centralized locations (such as Oxford) serving patients across regions, countries, or internationally.

## Methods

### Proposed Proof-of-Concept Projects

On the basis of the industry research, case studies, and local environment, we propose a spectrum of PoC projects that will create the capability to explore both cost-effectiveness, clinical efficacy, and user adoption of IoMT systems.

These projects cover 3 areas:

1. Bring Your Own Device Integration (BYOD) Integration,
2. Chronic disease management, and
3. Personal health records.

### Bring Your Own Device Integration

Bring your own device presents an opportunity for patients to use personal medical devices to capture health information. The purpose of this project will be to give patients the capability to use their own devices connected to the OUH EHR digital infrastructure for disease management.

#### Aims

The aims of this project will be as follows:

1. To enable EHR-integrated smart device interoperability,
2. To demonstrate accessibility to various devices across the same disease state, and
3. To compare use of personal devices with controlled medical devices and evaluate adoption and clinical data accuracy.

#### Technologies

The following technologies will be explored:

1. Cerner Care Aware activation and
2. Validic UK instance.

### Chronic Disease Management

The purpose of this project will be to evaluate the effectiveness of a mobile Health solution designed to provide accurate data collection and to create resource efficiency by replacing staff with technology for data capture and reporting.

#### Aims

The aims of this project will be as follows:

1. To evaluate impact to clinical staffing and hospital visits and
2. To evaluate patient perspectives on technology use.

#### Technologies

The following technologies will be explored:

1. Drayson gestational diabetes monitoring and
2. Cerner Care Aware activation.

### Adoption

The key considerations in the analysis of adoption are uptake and the sustained use of access to personal health records. This project will enable direct patient access of personal health records.

#### Aims

The aims of this project will be as follows:

1. To allow for access to patient record via disconnected device,
2. To create conduit for data exchange of recorded data via consumer device, and
3. To evaluate patient perspectives on technology use.

#### Technologies

The following technologies will be explored:

1. Cerner EHR and
2. Apple HealthKit.

### Proof-of-Concept Next Steps

It is recommended to hold a workshop over summer 2018 (late September), where this report can be presented to the Promoting the Internet of Things via Collaborations between Higher Education Institutions & Industry (PITCH-In) partners and projects can be scoped and developed in more detail.

Each project will first gather user needs to link needs to the underlying technology and then use an iterative process to conceptualize the overall solution, which will be beta-tested across an extended trial. Ethical approval will be sought for the study and evaluation design and each project stage-managed by a controlled project management process (Figure 1).

Projects will be expected to deliver 7 key outcomes:

1. Scoping document: project initiation document,
2. Evaluation approach including
  - Technical benchmarking of management strategy, security, governance, quality, operations, architecture, and supporting process,
  - Evaluation benchmarking of factors impacting adoption, including analysis of how socioeconomic, education, ethnicity, age groups, and associated factors are considered in take-up of technologies,
3. Needs analysis with patients,
4. Alpha prototype demonstrating,
5. Beta system implementation,
6. Completed evaluation, and
7. Final report.

## Results

This PoC projects will be funded by the Research England’s CCF. The project started in 2018. During March 2018, the OUH Digital Innovation Lead and Cooksey Fellows from the University of Oxford Health Care Translation Group attended the HIMSS 2018 conference and attended a site visit at Cerner’s Innovation campus. During this trip, the team investigated the potential for IoMT technologies and their applicability to the Oxford ecosystem. Results are expected to be available by the end of 2019.

## Discussion

### Internet Data Collection Enablement of Population Health Analysis

The rapidly developing Oxford digital health ecosystem offers tremendous opportunities for PoC studies to investigate and share understanding of the use of IoT in health care. During 2018 to 2019, several technical solutions will be deployed that will give Oxford a unique position within the United Kingdom (and only 1 of a small handful of international sites) to implement real-world health care IoT solutions. The combination of digital population health solutions to integrate data sources; patient portals to engage users; ingestion platforms for IoT devices; and open-source interoperability APIs can be leveraged to develop, test, and evaluate needs, ideas, and solutions with frontline staff, academics, and industry partners.

### Conclusions

This report has been prepared to support the University of Oxford’s input to the PITCH-In project, which is being led by the University of Sheffield in partnership with the universities of Oxford, Cambridge, and Newcastle and industrial and health sector partners. PITCH-In has been awarded £4.9 million by Research England’s CCF to drive forward collaboration concerned with the IoT across multiple industries, including health care.

## Abbreviations

AHSC: Academic Health Science Centre
AI: artificial intelligence
API: application programming interface
BYOD: Bring your own device
CCF: Connecting Capability Fund
CCG: Clinical Commissioning Group
CO: carbon monoxide
EHR: electronic health record
FHIR: Fast Health Care Interoperability Resources
GDE: Global Digital Exemplar
GP: general practice
IoMT: Internet of Medical Things
IoT: Internet of Things
HIMSS: Health Care Information and Management Systems Society
NHS: National Health Service
OCC: Oxford County Council
OH: Oxford Health
OUH: Oxford University Hospitals
PITCH-In: Promoting the Internet of Things via Collaborations between Higher Education Institutions & Industry
PoC: proof-of-concept
SCAS: South Central Ambulance Service
SMART: Substitutable Medical Apps, Reusable Technology
